# Progress in Photography

**Published:** 1892-11

**Authors:** 


### PROGRESS IN PHOTOGRAPHY.

A map of the heavens is being prepared at a cost of £1,200,000 (which the leading nations have agreed to raise), as an example of what telescope photography can do. Instantaneous photography seems to have attained perfection, for it is now possible to fix the image of a cannon ball flying through the air. Even microbes are now being photographed. A young French chemist, M. Henri Courtonne, is credited with a new discovery, for which we have been looking to Mr. Edison. Sound being transmissible by telephone, M. Courtonne argued by a rigorous analogy that light might be transmitted too. As the telephone consists of a transmitter, a wire and a receiver, so there was reason to believe that these three organs might be adapted for transmitting light vibrations, and for this purpose the transmitter and receiver should be prepared chemically for receiving and giving out light instead of sound vibrations. This was done by substituting sensitized photographic plates for the ordinary telephone plate. One of the plates was placed in front of an aperture through which an image was cast, and this image has been forwarded by wire and has been seen at the other end. The first apparatus was very imperfect, and M. Courtonne having heard that Mr. Edison was on the track of a similar discovery, resolved to publish his experiments, a description of which he, however, sent in a sealed letter to the Academy in 1889. This letter is only to be opened at the sender's request.

The *Figaro* says that the consequences of the telephotography cannot be over estimated. To-morrow, it says, you will see in Paris the image of a man smoking in St. Petersburg.—*London News*.

